# β-Hydroxy-β-methylbutyrate attenuates sepsis-associated lung injury by regulating NF-κB p65-mediated inflammation, ER stress and mitochondrial apoptosis in a rat model

**DOI:** 10.1007/s00210-026-05114-1

**Published:** 2026-02-19

**Authors:** Arif Timuroglu, Eyyup Sabri Ozden, Esma Selcuk, Emine Sarman, Furkan Cagri Oguzlar, Oznur Kolay, Halil Asci, Ulku Ceren Koksoy

**Affiliations:** 1 Department of Anesthesiology and Reanimation, Dr. Abdurrahman Yurtaslan Ankara Oncology Training and Research Hospital, University of Health Sciences, Ankara, Türkiye; 2 Faculty of Medicine, Department of Anesthesiology and Reanimation, Suleyman Demirel University, Isparta, Türkiye; 3 Faculty of Medicine, Department of Medical Biology, Suleyman Demirel University, Isparta, Türkiye; 4 Faculty of Medicine, Department of Histology and Embryology, Afyonkarahisar Health Sciences University, Afyonkarahisar, Türkiye; 5 Faculty of Medicine, Department of Emergency Medicine, Suleyman Demirel University, Isparta, Türkiye; 6 Faculty of Medicine, Department of Medical Pharmacology, Suleyman Demirel University, Isparta, Türkiye; 7 Faculty of Medicine, Department of Anesthesiology and Reanimation, Yuksek İhtisas University, Ankara, Türkiye

**Keywords:** Acute lung injury, Apoptosis, Endoplasmic reticulum stress, İnflammation, Mitochondrial stress

## Abstract

Sepsis-induced acute lung injury (ALI) is a leading cause of mortality in intensive care, driven by inflammatory, apoptotic, and oxidative stress pathways. β-Hydroxy-β-methylbutyrate (HMB), a leucine metabolite, exhibits anti-inflammatory and antioxidant effects, but its role in septic lung injury remains unclear. Thirty-two male Wistar rats were randomized into four groups (*n* = 8 each): Control, HMB (300 mg/kg), cecal ligation and puncture (CLP), and CLP + HMB. Lung tissues were analyzed histopathologically, nuclear factor kappa B p65 (NF-κB p65) and caspase 3 (Cas-3) expression was evaluated immunohistochemically, and mRNA expression of C/EBP homologous protein (CHOP), glucose-regulated protein 78 (GRP78), caspase 12 (Cas-12), B-cell lymphoma 2 (BCL-2), BCL-2-associated X protein (BAX), cytochrome C (Cyt-C), nuclear factor erythroid 2–related factor 2 (NRF2), and glutathione peroxidase 4 (GPX4) was measured at the molecular level. CLP induced upregulation of NF-κB p65, ER stress markers (CHOP, GRP78, Cas-12), and mitochondrial apoptotic proteins (BAX, Cyt-C, Cas-3), while downregulating BCL-2, NRF2, and GPX4 expression. HMB treatment reversed these expression changes and improved lung histopathology. HMB protects lungs in experimental sepsis by inhibiting NF-κB inflammation, reducing ER and mitochondrial apoptosis, and boosting antioxidant defenses via NRF2/GPX4. These findings support its potential as adjunct therapy for sepsis-induced ALI.

## Introduction

Sepsis is a life-threatening syndrome characterized by a dysregulated host response to infection, frequently leading to multiple organ dysfunction and high mortality worldwide (Singer et al. 2016). Among the most severely affected organs, the lung is particularly vulnerable; sepsis-associated acute lung injury (ALI) and acute respiratory distress syndrome (ARDS) represent major causes of hypoxemia, structural pulmonary damage, and poor outcomes in critical care practice (Bellani et al. 2016; Swenson and Swenson 2021). The pathophysiology of septic lung injury is complex, involving disruption of the alveolar–capillary barrier, interstitial edema, vascular congestion, and infiltration of inflammatory cells, ultimately leading to profound impairment of gas exchange and tissue integrity (Swenson and Swenson 2021; Ma et al. 2025; Zheng et al. 2019).

At the molecular level, multiple signaling cascades orchestrate the progression of sepsis-induced ALI. Extensive research, particularly in lipopolysaccharide (LPS)-stimulated sepsis models, has highlighted that targeting inflammatory signaling and oxidative stress is critical for mitigating lung injury (Zhang et al. 2020a, b, c). Nuclear factor kappa B (NF-κB) mediated pro-inflammatory responses, including the upregulation of tumor necrosis factor alpha (TNF-α), interleukin 1 beta (IL-1β), and interleukin 6 (IL-6), aggravate lung injury by promoting cytokine release and immune cell infiltration (Ma et al. 2025; Zheng et al. 2019; Sun et al. 2023). Apoptotic markers such as caspase 3 (Cas-3), B-cell leukemia/lymphoma 2 protein (BCL-2), BCL-2-associated X protein (BAX), and cytochrome C (Cyt-C), together with endoplasmic reticulum stress markers including C/EBP homologous protein (CHOP), glucose-regulated protein 78 (GRP78), and caspase 12 (Cas-12), contribute to alveolar epithelial and endothelial damage. Moreover, inadequate activation of nuclear factor erythroid 2-related factor 2 (NRF2)-dependent antioxidant pathways (such as glutathione peroxidase 4 (GPX4)) exacerbates oxidative stress, further amplifying tissue damage (Zheng et al. 2019; Sun et al. 2023; Li et al. 2019). Both clinical and experimental data emphasize that restoring the balance among these inflammatory, apoptotic, and oxidative pathways is critical for limiting the severity of lung injury in sepsis (Ma et al. 2025; Li et al. 2014, 2025; Bezerra et al. 2023).

**Fig. 1:**
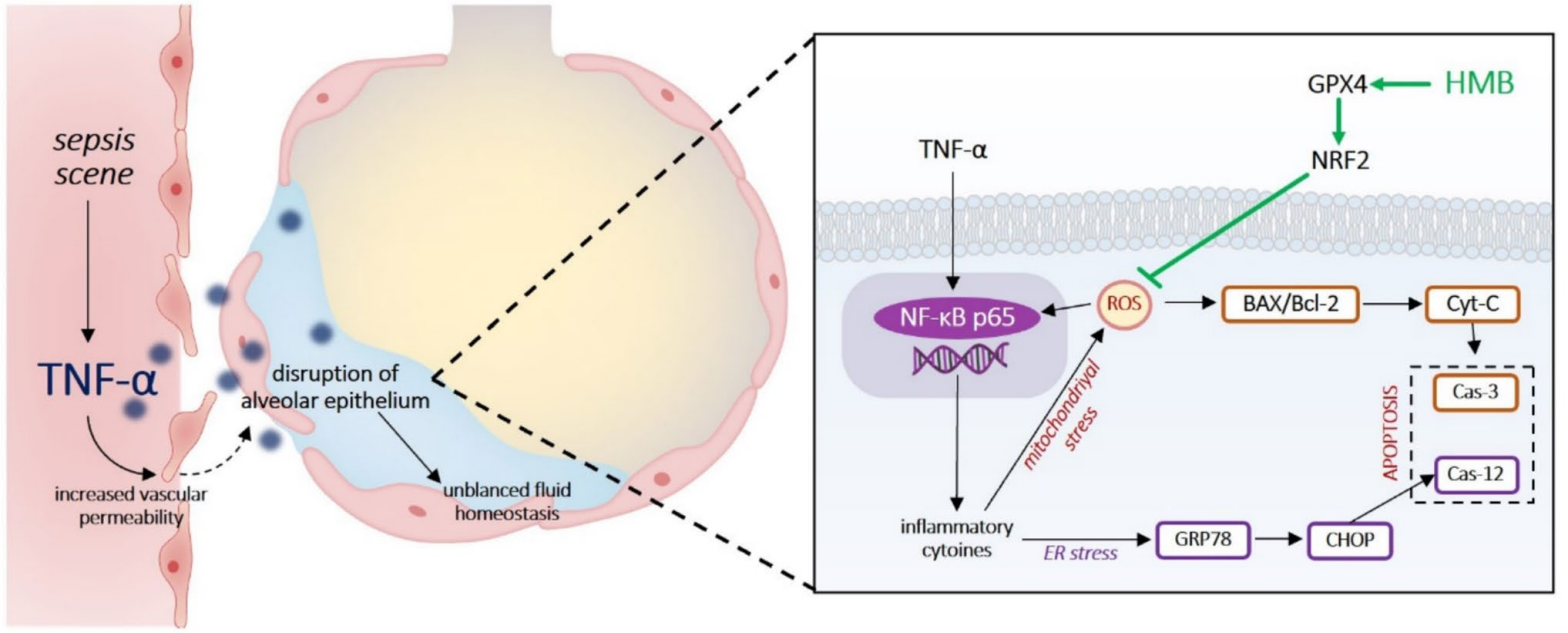
Mechanisms of alveolar injury in sepsis and the potential cytoprotective effect of HMB. *In sepsis, elevated TNF-α increases vascular permeability, leading to alveolar epithelial damage and pulmonary edema. TNF-α activates NF-κB p65 signaling, promoting proinflammatory cytokine production, which enhances ROS generation and triggers apoptosis *via* both mitochondrial (Bax/Bcl-2 imbalance, Cyt-C release, Cas-3 activation) and ER stress pathways (GRP78, CHOP, Cas-12). ROS can further amplify inflammation and ER stress. HMB mitigates oxidative stress and apoptosis by activating NRF2–GPX4 signaling and suppressing ROS production. CHOP: C/EBP homologous protein, GRP78: glucose-regulated protein 78, NRF2: Nuclear factor erythroid 2–related factor 2, Cas-12: Caspase 12, Cas-3: Caspase 3, BAX: BCL-2-associated X protein, GPX4: Glutathione peroxidase 4, BCL-2: B-cell leukemia/lymphoma 2 protein, TNF-α: Tumor necrosis factor alpha, NF-κB p65: Nuclear factor kappa B p65, ER: Endoplasmic reticulum, ROS: Reactive oxygen species, HMB: β-Hydroxy-β-methylbutyrate. Cyt-C: Cytochrome C*

β-Hydroxy-β-methylbutyrate (HMB), a metabolite of the essential amino acid leucine, has emerged as a candidate therapeutic agent due to its immunomodulatory, anti-inflammatory, anti-apoptotic, and antioxidant properties. Evidence from critical care and exercise/stress models has demonstrated that HMB reduces inflammation and oxidative stress, suppresses protein catabolism, and improves functional outcomes (Hsieh et al. 2006; Supinski and Callahan 2014; Arazi et al. 2019). In sepsis models, HMB has been shown to attenuate proteasome activation and Cas-3–mediated degradation, while preserving muscle function (Supinski and Callahan 2014). Clinical meta-analyses further suggest potential benefits of HMB supplementation in critically ill patients (Arazi et al. 2019). Collectively, these findings indicate that HMB may confer protective effects beyond skeletal muscle, potentially attenuating sepsis-associated lung injury through modulation of inflammatory and stress-related pathways (Fig. 1).

This study aimed to assess the protective effect of HMB on lung tissue in a CLP-induced sepsis model. Analyses focused on evaluating the transcriptional expression and tissue localization of markers related to inflammation (NF-κB), apoptosis (Cas-3, BAX, BCL-2, Cyt-C), and oxidative/ER stress (NRF2, GPX4, CHOP, GRP78, Cas-12) to determine HMB’s regulatory role in septic lung injury.

## Materials and methods

### Animals and experimental design

Thirty-two adult male Wistar Albino rats (250–350 g) were obtained from the Experimental Animal Research Center of Süleyman Demirel University. The study protocol was approved by the Suleyman Demirel University Local Animal Ethics Committee (Ethics No: 18.09.2025/09/629) and conducted in compliance with ARRIVE 2.0 guidelines.

Animals were housed under controlled conditions (21–22 °C, 55–60% humidity, 12-h light/dark cycle) with free access to standard chow and water. After one week of acclimatization, rats were randomly divided into four groups (*n* = 8): the Sham group received 1 mL of saline by oral gavage once daily for seven consecutive days, and on the final day, laparotomy was performed without inducing cecal ligation and puncture (CLP). The CLP group was treated with the same saline regimen and underwent the CLP procedure on day seven to induce sepsis (Chen et al. 2015). The CLP + HMB group received 300 mg/kg of HMB (BulkSupplements HMB Powder, Nevada, USA) solution orally for seven days, after which the CLP procedure was performed. This specific dosage (300 mg/kg) was selected based on previous studies demonstrating its efficacy in attenuating protein degradation and modulating inflammatory responses in rodent models of catabolic stress, without inducing toxicity (Supinski and Callahan 2014; Kusabbi et al. 2015). The HMB group was administered 300 mg/kg HMB orally for seven consecutive days and, on the final day, underwent laparotomy without CLP induction.

Twenty-four hours after surgery or sham procedure, rats were deeply anesthetized with ketamine (90 mg/kg, Keta-Control, Doğa İlaç, Türkiye) and xylazine (10 mg/kg, Xylazinbio, Bioveta, Czech Republic), then euthanized by exsanguination. Right lungs were fixed in 10% buffered formalin for histopathological and immunohistochemical analysis, while the remaining tissues were stored at − 80 °C for molecular assays.

### CLP procedure

The CLP model was used to induce polymicrobial sepsis with minor modifications. Rats were anesthetized with ketamine (90 mg/kg, Keta-Control, Doğa İlaç, Türkiye) and xylazine (10 mg/kg, Xylazinbio, Bioveta, Czech Republic), and a 2-cm midline laparotomy was performed. The cecum was ligated below the ileocecal valve with 4–0 silk, punctured once with an 18-gauge needle to release a small amount of feces, then repositioned and closed in two layers. Sham rats underwent surgery without ligation or puncture. All animals received warm saline and were monitored for signs of sepsis.

### Histopathological evaluation

Lung tissue samples were fixed in 10% buffered neutral formalin solution. They were subjected to routine histologic follow-up, embedded in paraffin, sectioned at 5-µm thickness and stained with hematoxylin–eosin. Histopathologic changes were examined at 400 × magnification in 20 randomly selected areas. Representative photomicrographs were captured at 200 × magnification (scale bar = 100 µm). In the staining evaluation, changes such as epithelial separation and disruption, thickening and congestion of the alveolar epithelium were scored between 0 and 4 (0: no damage; 1: damage in 25% of the area; 2: damage in 50% of the area; 3: damage in 75% of the area; 4: damage in 100% of the area) (Ozmen et al. 2024).

### Immunohistochemical evaluation

Paraffin block sections of 5-µm thickness were kept overnight in an oven at 45 °C and then stained using the streptavidin–biotin complex peroxidase method to evaluate Cas-3 and TNF-α expression. After Ultra V Block application, NF-kB p65 (ab32536, Abcam, 1:200) and Cas-3 (sc-56053, Santa Cruz, 1:200) primary antibodies were applied and incubated for 60 min. The sections were then treated with streptavidin-HRP and DAB (3,3′-diaminobenzidine) solution, stained with Harris hematoxylin and covered with entellan.

All preparations were evaluated under an Eclipse E-600 Nikon photomicroscope (Nikon, Japan) equipped with an image analysis system (NIS Elements, Nikon, Japan). Expression levels were semiquantitatively evaluated as follows: 0 (no staining), 1 + (weak staining), 2 + (moderate staining) and 3 + (strong staining) (Nurlu Temel et al. 2023).

### Real-time reverse transcription polymerase chain reaction (RT-qPCR)

Using the manufacturer’s protocol, RNA was isolated from homogenized tissues with the GeneAll RiboEx (TM) RNA Isolation Kit (GeneAll Biotechnology, Seoul, Korea). The amount and purity of the RNAs obtained were measured with the BioSpec-nano nanodrop (Shimadzu Ltd. Kyoto, Japan) device. 1 µg of RNA was used for cDNA synthesis. cDNA synthesis was carried out using the A.B.T. ™ cDNA Synthesis Kit (Atlas Biotechnology, Türkiye) in a thermal cycler according to the protocol. Primer designs were made by detecting specific mRNA sequences and testing possible primer sequences using the NCBI website. The primer sequences used are shown in Table 1. Expression levels of genes were measured in a Biorad CFX96 (California, USA) real-time PCR instrument using 2X SYBR green master mix (Nepenthe, Kocaeli, Türkiye). In the study, the beta actin gene was used as a housekeeping gene. The reaction mixture was prepared according to the manufacturer’s protocol to a final volume of 20 µl. The resulting reaction mixture was placed in a real-time qPCR device, and the cycling conditions were set according to the kit manufacturer’s protocol, and each sample was analyzed in triplicate. PCR conditions were as follows: an initial denaturation step at 94 °C for 10 min (1 cycle), followed by 40 cycles of denaturation at 95 °C for 15 s and combined annealing/extension at 57 °C for 30 s. Relative mRNA levels were calculated by applying the 2^− ΔΔCt^ formula to the normalized results (Imeci et al. 2025).

**Table 1:** Primary sequences, product size and accession numbers of genes

Genes	Primary sequence	Product size	Accession number
ACTB (HouseKeeping)	F: CCCGCGAGTACAACCTTCTT	481 bp	NM_031144.3
	R: AACACAGCCTGGATGGCTAC		
CHOP	F: TGGAAGCCTGGTATGAGGATCTG	175 bp	XM_006241445.4
	R: GAGGTGCTTGTGACCTCTGCTG		
GRP78	F: CCAATGACCAAAACCGCCTG	244 bp	NM_013083.2
	R: TGGCTTTCCAGCCATTCGAT		
Cas-12	F: CTGCATCAGAATCCAGGGGA	212 bp	NM_130422.1
	R: TCGGCCTTCCTTCTCCATCA		
NRF2	F: GCCTTCCTCTGCTGCCATTAGTC	126 bp	NM_001399173.1
	R: TCATTGAACTCCACCGTGCCTTC		
BAX	F: CACGTCTGCGGGGAGTCAC	419 bp	NM_017059.2
	R: TAGAAAAGGGCAACCACCCG		
GPX4	F: CATTGGTCGGCTGCGTGA	276 bp	NM_017165.4
	R: GGTTTTGCCTCATTGCGAGG		
BCL-2	F: CATCTCATGCCAAGGGGGAA	284 bp	NM_016993.2
	R: TATCCCACTCGTAGCCCCTC		
Cyt-C	F: TAAATATGAGGGTGTCGC	192 bp	NM_012839.2
	R: AAGAATAGTTCCGTCCTG		

*F* Forward,* R* Reverse, *ACTB* Beta Actin, *CHOP* C/EBP homologous protein, *GRP78* Glucose-regulated protein 78, *NRF2* Nuclear factor erythroid 2–related factor 2, *Cas-12* Caspase 12, *BAX* Bcl-2-associated X protein, *GPX4* Glutathione peroxidase 4, *BCL-2* B-cell leukemia/lymphoma 2 protein,* Cyt-C* Cytochrome C

### Statistical analysis

Statistical analyses were conducted using GraphPad Prism 10.1 (GraphPad Software, USA). Data normality was assessed with the Shapiro–Wilk test, and normally distributed results (*p* > 0.05) were expressed as mean ± SD. Group differences were analyzed by one-way ANOVA followed by Tukey’s post hoc test, with *p* < 0.05 considered significant.

Power analysis (G*Power 3.1) estimated a minimum of 28 rats (7 per group) for a large effect size (f = 0.40), α = 0.05, and power = 0.80. To enhance reliability and account for possible mortality, 32 rats (8 per group) were used.

## Results

### Histopathological findings

**Fig. 2:**
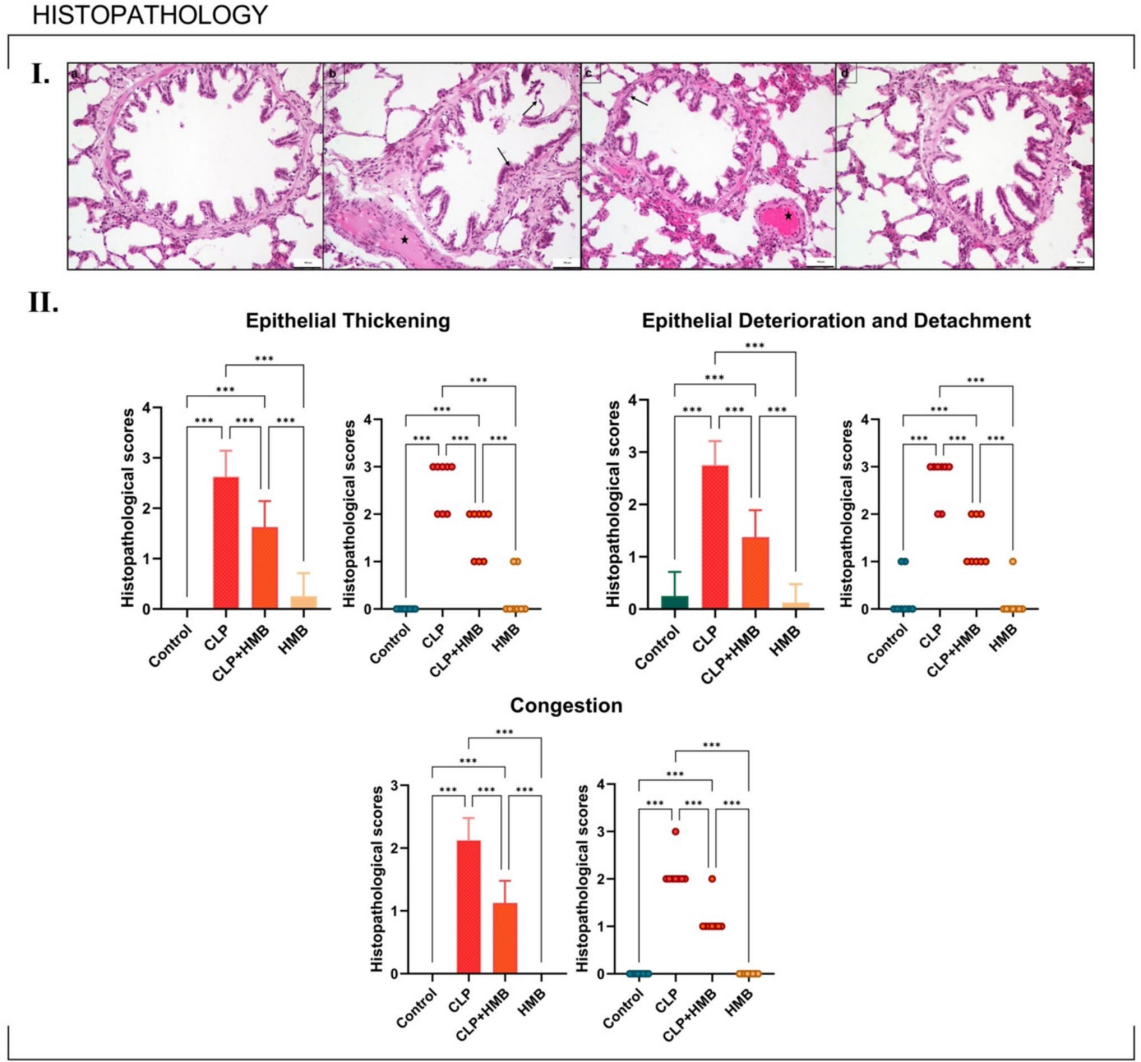
Representative H&E stained micrographs of lung tissues from all groups and graphical distribution of histopathological scores. *I: Normal lung histology was observed in the control (a) and HMB (d) groups. In the CLP group (b), detachment and structural degeneration of the bronchiolar epithelium (arrows), epithelial thickening, and marked congestion (asterisks) were evident. In the CLP* + *HMB group (c), epithelial damage and congestion were notably reduced. II: The bottom panel presents semi-quantitative scoring results. Epithelial thickening, epithelial detachment, and congestion were significantly higher in the CLP group compared with the control group (p* < *0.001), whereas these values were markedly decreased in the CLP* + *HMB group (p* < *0.001 vs. CLP). Scale bar* = 100 µm. Statistical analysis was performed by one-way ANOVA followed by Tukey’s test. ***p < *0.001. HMB: β-Hydroxy-β-methylbutyrate, CLP: Cecal ligation and puncture, H&E: Hematoxylin and eosin*

In the control and HMB groups, lung tissue maintained normal histological architecture. In the CLP-induced sepsis group, marked detachment and degeneration of the bronchiolar epithelium, epithelial thickening, interstitial edema, and congestion were observed. These findings indicate that the alveolar barrier integrity was disrupted, leading to fluid imbalance and tissue injury in the sepsis model. In the CLP + HMB group, these lesions were markedly reduced, and the bronchiolar structures and alveolar spaces appeared largely normal. Semi-quantitative scoring analysis demonstrated significantly higher levels of epithelial thickening, epithelial detachment, and congestion in the CLP group compared with the control group (*p* < 0.001). Treatment with HMB significantly improved these parameters (*p* < 0.001) (Fig. 2).

### Immunohistochemical findings

**Fig. 3:**
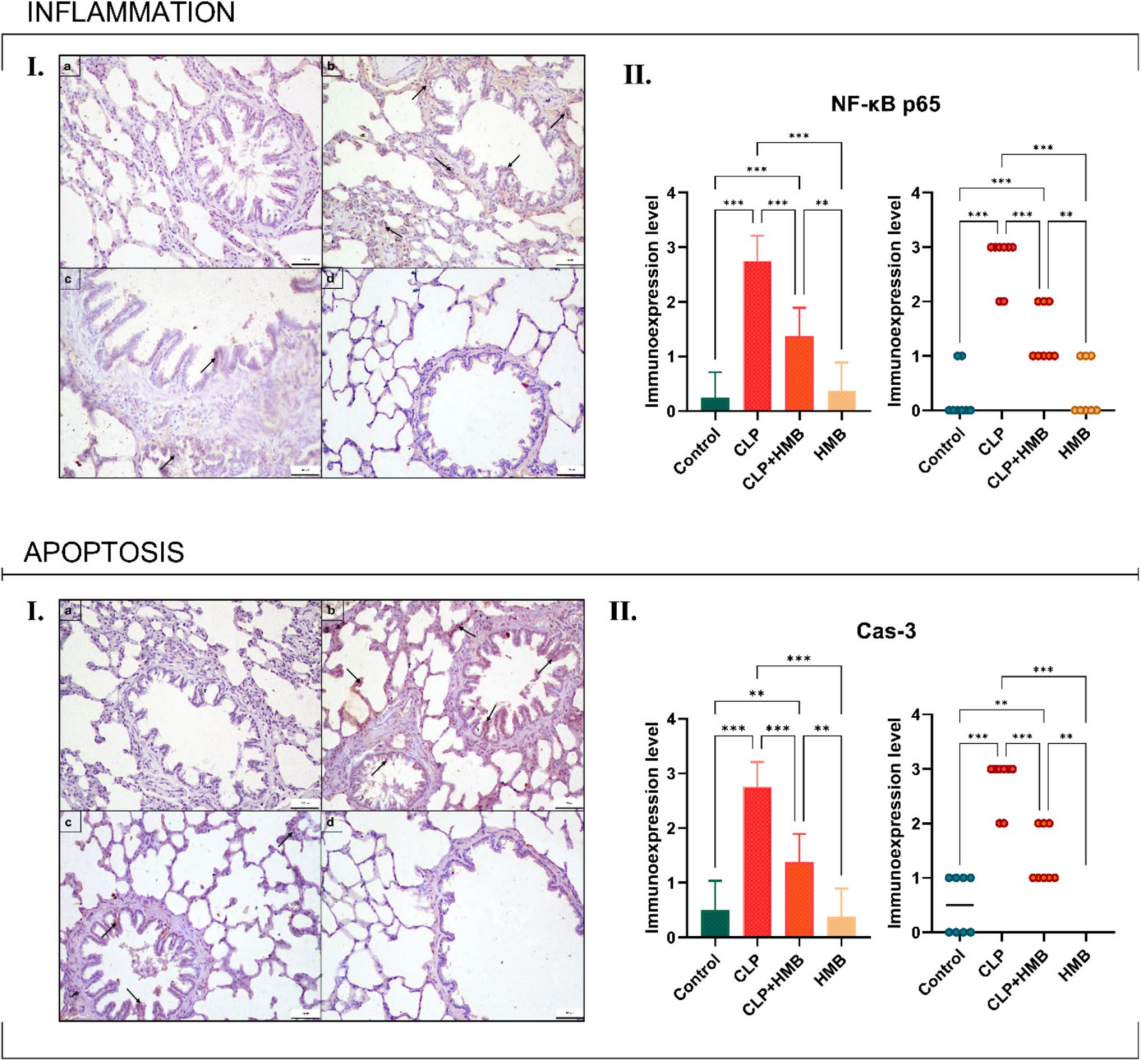
Immunohistochemical staining and quantitative scoring of NF-κB p65 and Cas-3 expression levels in tissue sections.* I: Weak expression was observed in the control (a) and HMB (d) groups. In the CLP group (b), intense NF-κB p65 and Cas-3 positivity (arrows) was detected in the alveolar and bronchiolar epithelium. In the CLP* + *HMB group (c), both expressions were significantly decreased. II: Quantitative analysis revealed a significant increase in the CLP group (p* < *0.001 vs control for both) and a marked reduction in the CLP* + *HMB group (p* < *0.001 vs. CLP for both). Scale bar* = 100 µm. Statistical analysis was performed by one-way ANOVA followed by Tukey’s test. ***p < 0.001, **p < *0.01. HMB: β-Hydroxy-β-methylbutyrate, CLP: Cecal ligation and puncture, NF-κB p65: Nuclear factor kappa B p65, Cas-3: Caspase 3*

Immunohistochemical analysis showed weak NF-κB p65 expression in the control and HMB groups. In the CLP group, a marked increase in NF-κB p65 expression was observed in the alveolar and bronchiolar epithelium (*p* < 0.001). In the CLP + HMB group, NF-κB p65 expression was significantly reduced (*p* < 0.001), demonstrating that HMB suppressed the inflammatory response (Fig. 3). Cas-3 expression, an executioner enzyme of apoptosis, was low in the control and HMB groups. In the CLP group, strong Cas-3 staining was detected in the alveolar and bronchiolar regions (*p* < 0.001). In the CLP + HMB group, Cas-3 expression was significantly decreased (*p* < 0.001), showing that HMB inhibited the apoptotic process (Fig. 3).

### Genetic findings

In the evaluation of ER stress markers, the CLP group showed significantly elevated mRNA levels of CHOP, GRP78, and Cas-12 compared with the control group (*p* < 0.001 for all). These molecules indicate ER stress and ER-mediated apoptosis. In the CLP + HMB group, expression of these genes was significantly reduced (*p* < 0.001 for all) compared to the CLP group, demonstrating that HMB attenuated ER stress–related injury.

**Fig. 4:**
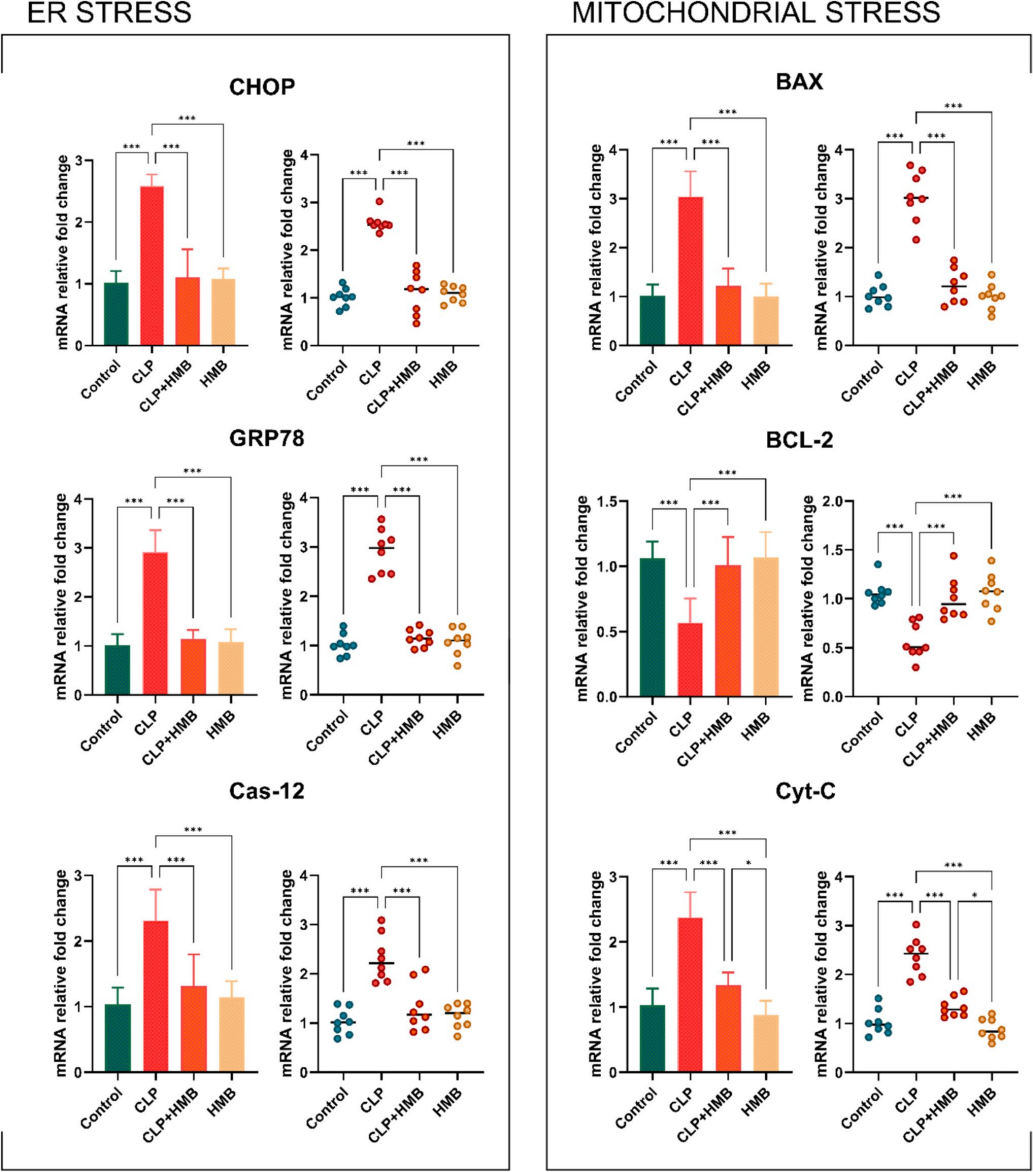
mRNA expression levels of ER stress-related and mitochondrial stress genes. *In the CLP group, CHOP, GRP78, and Cas-12 expression levels were significantly increased compared with the control group (p* < *0.001), whereas these values were markedly reduced in the CLP* + *HMB group (p* < *0.001 vs. CLP). These findings demonstrate that apoptosis was activated through the ER pathway in the sepsis model, and that HMB suppressed these mechanisms. Proapoptotic BAX and Cyt-C expression levels were elevated in the CLP group, while antiapoptotic BCL-2 was decreased (p* < *0.001 for all vs control). HMB treatment significantly reduced BAX and Cyt-C levels while increasing BCL-2 expression (p* < 0.001 for all vs CLP). These findings demonstrate that apoptosis was activated through the mitochondrial pathway in the sepsis model, and that HMB suppressed these mechanisms. Statistical analysis was performed by one-way ANOVA followed by Tukey’s test. ***p < 0.001, *p < *0.05. HMB: β-Hydroxy-β-methylbutyrate, CLP: Cecal ligation and puncture, CHOP: C/EBP homologous protein, GRP78: Glucose-regulated protein 78, Cas-12: Caspase 12, ER: Endoplasmic reticulum, BAX: BCL-2-associated X protein, BCL-2: B-cell leukemia/lymphoma 2 protein, Cyt-C: Cytochrome C*

Analysis of mitochondrial stress and apoptotic pathways revealed a marked increase in the expression of the proapoptotic markers BAX and Cyt-C in the CLP group compared to the control (*p* < 0.001 for both), while antiapoptotic BCL-2 expression was significantly decreased (*p* < 0.001). These findings indicate activation of mitochondrial apoptosis in the sepsis model. In the CLP + HMB group, BAX and Cyt-C expression levels were significantly reduced, whereas BCL-2 expression was increased compared to the CLP group (*p* < 0.001 for all), showing that HMB inhibited mitochondria-dependent apoptosis (Fig. 4).

**Fig. 5:**
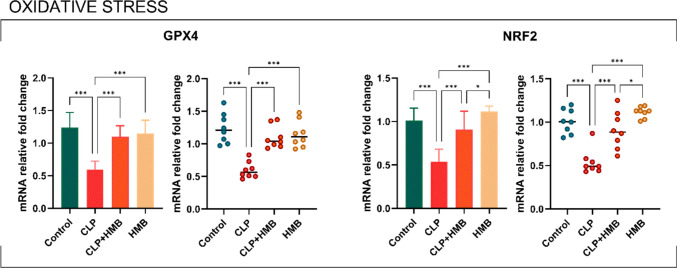
mRNA expression levels of oxidative stress parameters. *In the CLP group, expression of GPX4, one of the key antioxidant defense enzymes, was decreased (p* < *0.001 vs. control). In the CLP* + *HMB group, GPX4 expression was increased (p* < *0.001 vs CLP), indicating that HMB alleviated oxidative stress. NRF2 levels were reduced in the CLP group, while a significant recovery was observed in the CLP* + HMB group. Statistical analysis was performed by one-way ANOVA followed by Tukey’s test. ***p < 0.001, *p < *0.05. HMB: β-Hydroxy-β-methylbutyrate, CLP: Cecal ligation and puncture, NRF2: Nuclear factor erythroid 2–related factor 2, GPX4: Glutathione peroxidase 4*

Regarding oxidative stress markers, the CLP group showed reduced expression of GPX4, a key antioxidant enzyme (*p* < 0.001). In the CLP + HMB group, GPX4 expression was significantly increased (*p* < 0.001), supporting the antioxidative effect of HMB. NRF2, a regulatory transcription factor of the antioxidant response, was detected at low levels in the CLP group (*p* < 0.001). Significant recovery was observed in the CLP + HMB group compared to the CLP group (*p* < 0.001) (Fig. 5).

## Discussion

Sepsis is a life-threatening syndrome characterized by a dysregulated host response to infection, frequently progressing to multiple organ failure and high mortality. Among the affected organs, the lungs are particularly vulnerable due to their extensive vascular network and exposure to circulating inflammatory mediators (Singer et al. 2016). This often culminates in ALI and ARDS (Bellani et al. 2016). These complications prolong mechanical ventilation and increase mortality in septic patients. Therefore, therapeutic approaches capable of attenuating sepsis-related lung injury are of substantial clinical importance.

In the present study, we investigated the effects of HMB on lung tissue in a CLP-induced sepsis model. The pathophysiology of sepsis involves multiple mechanisms, including inflammation, apoptosis, oxidative stress, and ER stress, all of which were consistent with the alterations observed in our model (Fang et al. 2020; Sahoo et al. 2025). Consistent with extensive research on lipopolysaccharide (LPS)-induced sepsis models (Zhang et al. 2020a, b, c), our findings confirm that targeting these pathways is essential for organ protection. In the CLP group, typical histopathological findings such as epithelial detachment, edema, and congestion were prominent, whereas HMB treatment significantly ameliorated these changes. This aligns with reports highlighting the critical role of alveolar–capillary barrier integrity in determining sepsis-related mortality (Ziaka and Exadaktylos 2025). Previous animal studies have shown that HMB prevents sepsis-induced diaphragmatic weakness and attenuates skeletal muscle proteolysis (Arazi et al. 2019; Kovarik et al. 2010). Our findings provide evidence that these protective properties are not restricted to muscle tissue but also extend to mitigating lung injury in a polymicrobial sepsis model.

Mechanistically, our transcriptional and immunohistochemical analyses indicated that HMB treatment coincided with suppressed NF-κB p65 expression and Cas-3 levels in septic lungs. NF-κB is a central transcription factor that regulates the expression of proinflammatory cytokines, while Cas-3 is the final executor of apoptotic pathways (Li et al. 2025; Wier et al. 2015). Their simultaneous downregulation suggests that HMB may modulate the crosstalk between inflammation and apoptosis. Previous studies of sepsis-induced liver injury have similarly shown that HMB decreases Cas-3 activity (Duan et al. 2021).

Our study also demonstrated that CLP triggered ER stress, evidenced by increased mRNA expression of CHOP, GRP78, and Cas-12. HMB treatment significantly reduced these markers. The suppression of Cas-12 is particularly relevant, as this mediator directly activates Cas-3 independent of mitochondrial signaling, amplifying apoptotic injury (Dho et al. 2025). By inhibiting Cas-12 expression, HMB may interrupt this vicious cycle. These observations are consistent with findings from LPS-induced sepsis models, where pharmacological modulation of ER stress provided protection against ALI (Asci et al. 2025; Zhang et al. 2020a, b, c).

Although the exact molecular interactions remain to be fully elucidated, our findings allow for mechanistic speculation regarding how HMB exerts these protective effects. Regarding NF-κB inhibition, we postulate that HMB acts upstream of the NF-κB complex, potentially by inhibiting the phosphorylation and degradation of the inhibitory protein IκBα, thereby preventing the nuclear translocation of the p65 subunit. This mechanism aligns with previous reports demonstrating HMB’s ability to suppress the ubiquitin–proteasome pathway, which is required for IκBα degradation (Eley et al. 2008). Furthermore, in the context of ER stress, HMB appears to function as a chemical chaperone or an enhancer of the adaptive Unfolded Protein Response (UPR). By upregulating or stabilizing GRP78 (BiP), HMB may facilitate proper protein folding and prevent the hyper-activation of the PERK/ATF6/IRE1 sensors. This stabilization likely suppresses the downstream lethal arm of the UPR, specifically the CHOP and Caspase-12 pathway (Duan et al. 2016).

Furthermore, our results confirmed that CLP activated mitochondrial apoptosis, as indicated by increased BAX and Cyt-C expression and decreased BCL-2. HMB reversed these alterations, downregulating pro-apoptotic mediators and restoring anti-apoptotic defenses. Preservation of mitochondrial integrity is essential for maintaining energy balance and preventing excessive apoptosis (Bezerra et al. 2023; Hu et al. 2024). The ability of HMB to stabilize mitochondrial function in septic lungs is in line with previous evidence demonstrating its regulatory effects on mitochondrial activity and lipid metabolism in adipocytes (Duan et al. 2023).

Additionally, oxidative stress represents a pivotal mechanism of sepsis-induced ALI. In our model, CLP reduced GPX4 and NRF2 expression, reflecting impaired antioxidant defense. GPX4 is a critical enzyme that protects against lipid peroxidation and has been closely linked to the regulation of ferroptosis (Zhang et al. 2024). HMB supplementation significantly restored GPX4 levels, suggesting that it may counteract oxidative and ferroptotic pathways (Holeček 2017). Considering that oxidative stress not only causes direct tissue injury but also amplifies inflammation through NF-κB activation, the antioxidant properties of HMB likely act synergistically with its anti-inflammatory effects to protect lung tissue (Sun et al. 2023).

A notable observation in our study was the magnitude of molecular changes compared to histological findings.While H&E staining revealed moderate damage to the alveolar structure, qPCR data showed robust increases in stress markers (CHOP, Caspase-12, BAX). This discordance is likely attributable to the temporal dynamics of cellular injury; transcriptional changes (mRNA upregulation) are acute and highly sensitive early indicators of cellular stress that often precede the profound morphological damage visible on light microscopy. Therefore, the robust molecular response observed here represents the early activation of protective or apoptotic pathways before irreversible tissue destruction becomes widespread.

This study has certain limitations that should be addressed in future research. First, HMB was tested at a single dose (300 mg/kg). This dose was selected based on previous pharmacokinetic and efficacy studies in rodent models of catabolic stress (Supinski and Callahan 2014); however, in compliance with ethical efforts to reduce animal usage (3R), we prioritized a known effective dose rather than performing a large-scale dose–response analysis (Tannenbaum and Bennett 2015). Consequently, the minimum effective or optimal therapeutic dose remains to be determined. Second, the 24-h observation period limits our understanding of long-term survival and late-stage pulmonary fibrosis. Third, our mechanistic insights rely primarily on gene expression (qPCR) and tissue localization (IHC). While these methods consistently point towards the regulation of NF-κB and ER stress pathways, we did not perform Western blotting or use specific pathway inhibitors (e.g., NF-κB inhibitors) to confirm causality at the protein level. Despite these limitations, the convergence of histological improvement with transcriptional downregulation of key stress markers provides strong preliminary evidence for HMB’s therapeutic potential in septic lung injury.

## Conclusion

In summary, HMB provided marked protection against sepsis-induced ALI in the CLP model by reducing histopathological injury, suppressing NF-κB–driven inflammation, alleviating ER stress and apoptosis, and enhancing antioxidant defense via the NRF2/GPX4 pathway. These findings suggest that HMB’s cytoprotective effects extend to lung tissue and support its potential as a safe adjunctive therapy for sepsis, warranting further experimental and clinical evaluation.

## Data Availability

The datasets generated and/or analyzed during the current study are available from the corresponding author on reasonable request.
